# Companion Animals and Child/Adolescent Development: A Systematic Review of the Evidence

**DOI:** 10.3390/ijerph14030234

**Published:** 2017-02-27

**Authors:** Rebecca Purewal, Robert Christley, Katarzyna Kordas, Carol Joinson, Kerstin Meints, Nancy Gee, Carri Westgarth

**Affiliations:** 1 Institute of Infection and Global Health, and Institute of Veterinary Science, Faculty of Health and Life Sciences, University of Liverpool, Leahurst Campus, Neston, Cheshire CH64 7TE, UK; robc@liverpool.ac.uk (R.C.); carri.westgarth@liverpool.ac.uk (C.W.); 2 Department of Epidemiology and Environmental Health, University at Buffalo, 270 Farber Hall, Buffalo, NY 14214, USA; kkordas@buffalo.edu; 3 School of Social and Community Medicine, University of Bristol, 39 Whatley Road, Bristol BS8 2PS, UK; carol.joinson@bristol.ac.uk; 4 School of Psychology, University of Lincoln, Brayford Pool, Lincoln, Lincolnshire LN6 7TS, UK; kmeints@lincoln.ac.uk; 5 Department of Psychology, State University of New York, Fredonia, NY 14063, USA; nancy.gee@fredonia.edu; 6 WALTHAM Centre for Pet Nutrition, Waltham-on-the-Wolds, Melton Mowbray, Leics LE14 4RT, UK

**Keywords:** pet ownership, human-animal interaction, review, child development, adolescent development

## Abstract

Childhood and adolescence are important developmental phases which influence health and well-being across the life span. Social relationships are fundamental to child and adolescent development; yet studies have been limited to children’s relationships with other humans. This paper provides an evidence review for the potential associations between pet ownership and emotional; behavioural; cognitive; educational and social developmental outcomes. As the field is in the early stages; a broad set of inclusion criteria was applied. A systematic search of databases and grey literature sources found twenty-two studies meeting selection criteria. The review found evidence for an association between pet ownership and a wide range of emotional health benefits from childhood pet ownership; particularly for self-esteem and loneliness. The findings regarding childhood anxiety and depression were inconclusive. Studies also showed evidence of an association between pet ownership and educational and cognitive benefits; for example, in perspective-taking abilities and intellectual development. Evidence on behavioural development was unclear due to a lack of high quality research. Studies on pet ownership and social development provided evidence for an association with increased social competence; social networks; social interaction and social play behaviour. Overall, pet ownership and the significance of children’s bonds with companion animals have been underexplored; there is a shortage of high quality and longitudinal studies in all outcomes. Prospective studies that control for a wide range of confounders are required.

## 1. Introduction

Childhood and adolescence are crucial life phases in their contribution to the quality of health, emotional well-being, learning and behaviour across the life span [1]. Relationships with others are fundamental contributors to child and adolescent development according to relationship psychology [2] and attachment theory [3]. Yet, studies of child development have largely been limited to children’s relationships and interactions with other humans. However, animal ownership is common. Recent figures indicate that 68% of U.S. households [4] and 46% of British households [5] include at least one companion animal. Moreover, epidemiological studies suggest that pets are more likely to be found in households with children than in any other household type [6,7,8,9]. Although pet ownership and children’s bonds with companion animals may have the potential to positively influence child and adolescent development, these relationships have received little attention and a need for research in this area has been recognized [9,10]. Considering that pet ownership also pertains risks, such as zoonoses, bites and asthma/allergies [11], it is important that the impact of pet ownership on childhood development is investigated in detail. Interactions with animals may affect several aspects of human development: emotional, behavioural, cognitive, educational and social. 

Companion animals (including horses, dogs, cats, rabbits and other rodents) have the potential to promote healthy emotional youth development in many ways, as shown by research in Human-Animal Interactions (HAI) (the mutual and dynamic relationships between people and animals and the ways in which these interactions may affect physical and psychological health and well-being of both people and their pets [12]). This paper uses the term “youth” development to refer to all age ranges within Infancy (0–2 years), Early childhood (2–5 years), Later childhood (6–12 years) and Adolescence (13–18 years). There is growing evidence that children turn to their pets for comfort, reassurance and emotional support when feeling anger, sadness, or happiness [13,14,15,16]. Thus, it is plausible that companion animals may have the potential to encourage better emotional health and reduce anxiety and depression. Physiological mechanisms, such as activation of the oxytocin system may partly explain this reduction of psychological stress for humans who are in contact with animals [17]. However, it is important to recognize that pet attachment may be more important in exerting these potential effects than pet ownership. According to attachment theorists, when attachment behaviours are consistently met by the primary caregiver, children form secure internal working models (a cognitive framework consisting of mental representations for understanding the world, self and others) that are foundational for their ability to make affectionate bonds with others and to create and maintain close relationships [3]. Although psychological theories of attachment concentrate on attachment between humans, research has demonstrated that children display attachment behaviours towards their pets [18]. Because companion animals both give and receive affection, they can contribute to and partially fulfil attachment needs; therefore, the developmental importance of bonds that children and adolescents form with animals should not be overlooked [9,19]. In addition, children who develop poor parental attachment tend to nurture internal working models of distrust with others, insecurity, separation anxiety, low self-esteem, and a propensity for loneliness [20,21,22]. If children are able to develop secure attachment behaviours with their pets as a substitute, secure internal working models may still develop to some extent [23]. Whether pet attachment and ownership has any impact on child and adolescent development is currently unclear. 

Self-psychology (self-esteem, self-cohesion and self-acceptance) is another important aspect of youth development. Particularly in early and pre-adolescence, developmental changes in self-esteem have a significant impact and fluctuate prominently, with large decreases in self-esteem during transition to adolescence [24]. It has been suggested that if companion animals provide support for self-esteem, their greatest influence will be on youths as they approach adolescence (coinciding with increasing experiences of uncertainty) and at this time they may have a higher need for the emotional support they derive from companion animals [25]. Also, during this period cognitive changes in thinking about the self and others, as well as relationships with significant others, such as parents and peers (and perhaps pets), are most common and can indirectly affect self-esteem [25]. If companion animals provide social support [15] and act as catalysts for human social interactions [26], they may reduce loneliness and increase self-esteem. Companion animals have been found to rival and even surpass humans ability to provide important self-object needs, such as self-cohesion, self-esteem, calmness, soothing, and acceptance [27]. Increased self-esteem and self-worth may result in further benefits for individuals with anxiety, depression, behavioural problems and educational attainment. However, whether causality can be implied to a link between companion animals and child or adolescent self-psychology is yet unknown.

Companion animals may also influence cognitive development. It has been suggested that companion animal ownership may facilitate language acquisition and potentially enhance verbal skills in children [28]. This could occur as a result of the companion animal functioning both as a patient recipient of the young child’s babble and as an attractive stimulus, eliciting verbal communication from young children in the form of praise, orders, encouragement, and punishment [28]. In addition, although not empirically tested, the pet may also serve as a subject of conversations that stimulate vocabulary building, when caregivers and children talk about what the pet is doing. Melson [9] reports evidence that companion animals may stimulate a young child’s cognitive growth through curiosity and learning, while also providing emotional support and unconditional positive regard. Melson [9] stated that for many children, companion animals are likely to be powerful motivators for learning, perhaps due to children learning and retaining more about subjects they are more emotionally invested in, and due to learning being optimized when it occurs within meaningful relationships. The presence of animals has been shown to elicit immediate positive effects in testing situations of cognition such as memory, categorization and attention [29,30,31,32,33,34] and studies on language, literacy, and reading ability have also shown a similar positive influence of animal presence [35,36,37]. It has been speculated that animal interaction may provide opportunities to improve cognitive Executive Functions (EFs) (mental processes that form the basis for planning, attention, memory and self-control) through stress reduction and social support which in turn can affect behaviour and improve academic outcomes [38]. Thus it could be plausible that the long-term presence of pets at home will have tangible influences on children’s cognitive development and educational outcomes. However, the quality of the existing evidence has not yet been reviewed to infer any conclusions.

Most research to date addressing the impact of pets on human health has focused on adults. Less is known about the role pets play in the lives and wellbeing of children and youths, and if pet ownership may provide scaffolding in child development. As outlined above, there is theoretical potential for the role of pets in child and adolescent development, which suggests these relationships are worth exploring further. However, the existing evidence has not been systematically reviewed to identify particular strengths or gaps in knowledge, nor as to whether causality can be implied. Due to study design and quality this is a complex task.

Therefore the objective of this systematic review was to determine the evidence base for the impact of pet ownership and pet attachment on childhood and adolescent development. A broad range of outcomes were reviewed, including emotional, behavioural, cognitive, educational and social developmental. Recommendations for future research are provided to help advance the field of child development and HAI research.

## 2. Materials and Methods

Literature searches of journal articles published between 1960 and 2016 (as of 1 June 2016) were conducted in databases PsycINFO, CINAHL, PubMed, MEDLINE, Web of Science, ScienceDirect and grey literature sources.

Key terms used in searches included pet-related keywords (pet, pet ownership, dog, cat, dog ownership, companion animal, and human animal interaction) and were crossed with developmental-related keywords (child development, adolescent development, psychological, behavioural, educational, cognitive, language and social development, anxiety, depression, self-esteem, loneliness, emotional health). Websites on human-animal interaction were reviewed for possible research articles, including https://www.waltham.com/waltham-research/hai-research/ and https://habricentral.org/resources/browse/journalarticles. In addition, reference lists from relevant journal articles were scanned. It is still possible that evidence remains in unfound grey literature.

The inclusion criteria for the collection of articles included: literature that investigated the effects of pet ownership on emotional, cognitive or behavioural development in children and adolescents without developmental disabilities (infancy up to 18 years). Only articles written in English were included. With the aim of carrying out a broad review of the current relevant literature, restrictions for inclusion were limited; papers were not excluded based on study design and methodology. 

Initially, abstracts were reviewed for study selection by the primary author. Research excluded on the basis of content and deemed not relevant to the aim of this paper included Animal Assisted Therapy (AAT), therapy and classroom animals, pets and their effect on physical health (asthma/allergy or other chronic illnesses), ethical and moral development. 

The studies were then assessed by the primary author against the OCEBM (Oxford Centre for Evidence-Based Medicine) levels of evidence 2011 [39] to take into account the risk of bias and quality of evidence on which conclusions are based, although no study was excluded based on quality alone due to large gaps in current evidence and poor availability of good-quality studies within each outcome (refer to Table 1 and Table 2 for details of classification).

## 3. Results

**Figure 1:**
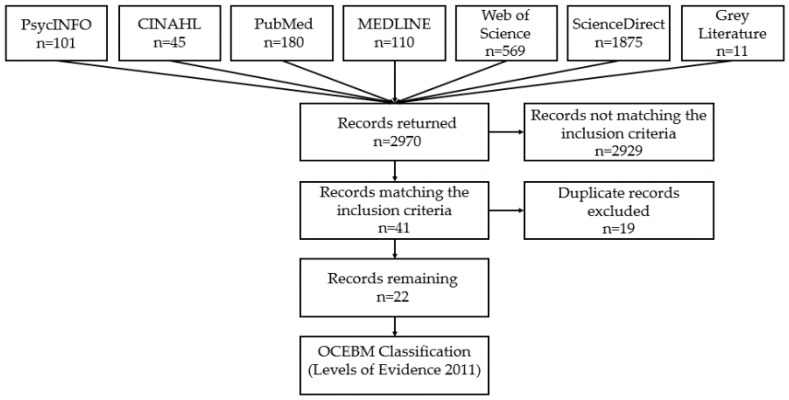
PRISMA (Preferred Reporting Items for Systematic Reviews and Meta-Analyses) flow diagram.

The initial literature searches returned 2959 results. Grey literature searches found an additional 11 references totalling 2970 publications (Figure 1). Forty-one publications remained after the examination of studies against the inclusion criteria. After removing duplicates and the studies not fitting the criteria, 22 studies remained for review.

Among the selected studies, which commonly reported on more than one outcome, 19 reported on the effects of pet ownership on emotional health, five on behavioural development, three on cognitive development, four on educational outcomes, and four on social development. Of the 22 studies, 13 reported cross-sectional data and only two reported longitudinal data on the impact of pets on youth development; a further one used mixed methods, and six qualitative studies were included.

**Figure 2:**
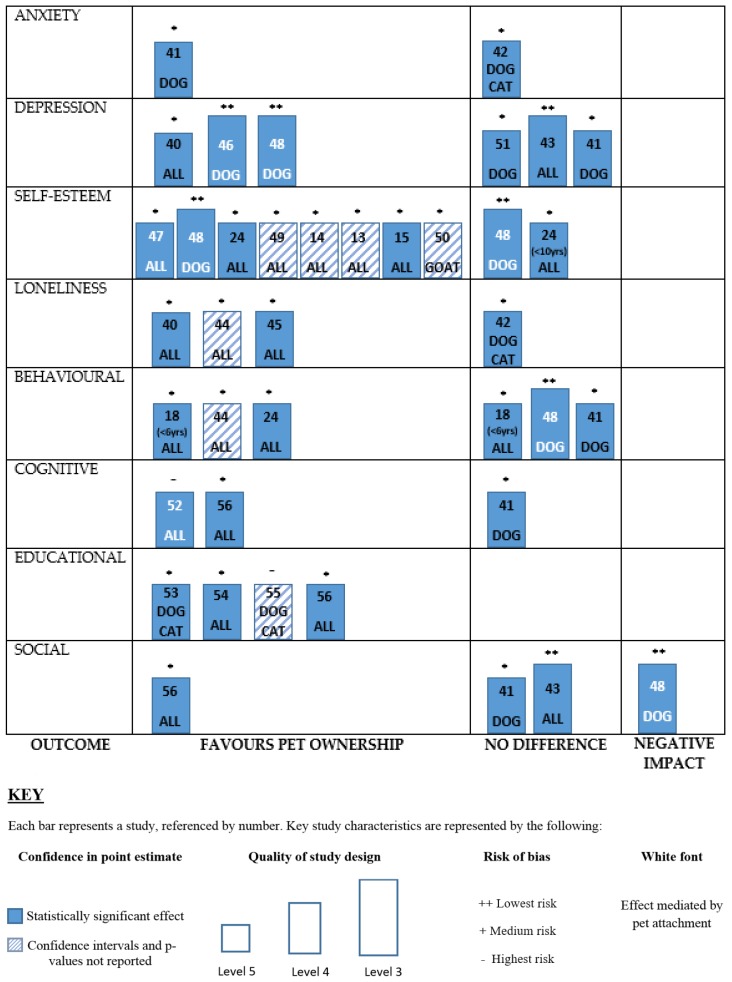
Harvest plot showing evidence for the impact pets have on categories of child and adolescent development. The table consists of eight rows (one for each dimension of development) and three columns (showing the differential effects of the evidence in each category). Each study is represented by a bar in each row; studies can be identified by reference number. Statistically significant effects (use of *p*-values) are indicated with solid blue bars, and studies with no confidence intervals and *p*-values reported are striped bars. The quality of study design is indicated by the height of the bar as categorised by OCEBM level of Evidence 2011. Each bar is annotated with marking to show risk of bias.

Bias was determined based on the Oxford Centre for Evidence-Based Medicine 2011 Levels of Evidence criteria [39]. OCEBM levels of evidence rankings were as follows: twenty papers were ranked level IV, and two papers were ranked at level III. Specific details of the literature can be found in Table 2. The majority of the studies were observational cross-sectional questionnaire surveys, or qualitative interviews, therefore were not further evaluated on their methodological quality as they are already considered low or very low levels of evidence according to OCEBM 2011. Refer to Figure 2 for a graphical representation of study design and risk of bias. Meta-analysis was not appropriate due methodological differences and the number of different outcomes reported.

### 3.1. Emotional Health Outcomes

Nineteen of the 22 studies were devoted to children’s emotional health. A wide range of emotional health benefits from childhood pet ownership were identified.

#### 3.1.1. Anxiety

Two studies measured anxiety as an outcome in youth pet ownership. Having a pet dog was associated with a decreased likelihood of general anxiety (12% of children with dogs met the clinical cut-off value for anxiety compared with 21% children without dogs) as measured by commonly used and validated mental health assessment tools, specifically Panic (“My child gets really frightened for no reason at all”), Separation Anxiety (“My child is afraid to be alone in the house”) and Social phobia/anxiety (“My child is shy”), in an American study of children aged 4–10-years in a paediatric primary care setting [41]. However, no evidence of a difference was found for Generalized Anxiety (“People tell me that my child worries too much”) and Significant School Avoidance (“My child is scared to go to school”). In contrast, in a Croatian study of 10–15-year-old children, pet owners (dog and cat) had no difference in validated social anxiety measures compared to non-pet owners [42]. In sum, these studies illustrate some potential of pet dogs to prevent child and adolescent anxiety, specifically separation and social anxiety disorders, but the small number of studies and mixed results warrant further research. Whether pets can reduce more general child anxiety is unknown.

#### 3.1.2. Depression

There is again a marked lack of research focusing on the effects of pet ownership on depressive symptoms in children and adolescents. Findings of the studies included in this review should be interpreted with caution; there is likely to be an indirect effect of pet ownership on depression, perhaps mediated by self-esteem or loneliness/social isolation.

In one study, pet owning homeless adolescents utilizing two Los Angeles drop-in centres reported fewer symptoms and lower average scores of self-reported depression measured by the 10-item Centre for Epidemiological Studies Depression Scale (CES-D) (average score of 7.8) in comparison to non-pet owning peers (10.2) [40]. However, data from an Australian school-based population study show pet-owning youths of similar ages (13–19 years) did not have better self-reported emotional health or well-being, suggesting findings may be different in non-homeless youths [43].

The potential protective effects of pets may also differ by age group. Prospective research in 8–12-year-olds found that high levels of attachment to a pet dog were negatively associated with maternal reports of tearfulness and weepiness at a 12 months follow up (*p* < 0.01) [48]. However, the impact of dog ownership on depressive symptoms in younger children measured by the Pediatric Symptom Checklist 17 (internalizing symptoms subscale) showed no significant effects, and in addition no difference was found between dog-owning and non-dog-owning children in their histories of diagnosed mental health problems [41]. Therefore it could be speculated that the relationship with the animal may be of more importance in conferring psychological benefits than pet ownership alone.

#### 3.1.3. Self-Esteem

Nine studies investigated the impact of pets on the self-esteem and self-concept among youths. No effect on self-esteem was found in pet-owning war-traumatized children (11–15 years) in Croatia using the Croatian Version of Rosenberg’s Self Esteem Scale (SES) [46]. In the same study, the type of pet owned had no effect either on validated self-esteem measures. In a different study of school children aged 9–18 years, children’s attachment to pets mediated the relationship between self-esteem as measured using validated self-report measures [47]. Therefore, there may be a relationship between the level of attachment to one’s pet and self-esteem benefits accrued. In addition, prospective research found (using maternal reported data) that higher levels of children’s (8–12 years) attachment to a pet dog were positively associated with changes in their confidence level (*p* < 0.005) over a 6 months period [48].

In contrast, in a mixed-methods study of children aged 10–13 years, pet owners in fifth (m = 16.7) and sixth grade (m = 17.2) reported higher levels of self-esteem than non-pet owners (m = 20.0, m = 20.8) (lower mean indicative of greater self-esteem) (*p* < 0.04) and pet owning sixth graders had higher self-concept scores in comparison to non-pet owners in the same grade (pet owners: m = 94.2, non-pet owners: m = 83.2) (*p* < 0.001) [25], even though greater attachment to pets was not related to self-esteem or self-concept. However, in the same study, children aged 8–10 did not differ in terms of self-esteem compared to non-pet owners, suggesting that pets exert their greatest influence during pre-adolescence and adolescence [25]. Other studies also indicate that pet ownership alone is sufficient to have a positive effect on self-esteem or self-concept, independent of pet attachment. Among 8–13-year-olds, qualitative research supports the finding companion animals increase child and adolescents self-esteem and self-enhancing affection—the perception that the child-pet relationship imparts a sense of self-importance and makes them feel good about themselves [16]. Further qualitative data supports this. In a study of 7–8-year-old children examining representations of social support from companion animals using a story-based methodology, relationships with pets were ranked higher than human relationships by children as providers of both self-esteem and support [15]. Generally, dogs and cats were deemed better providers of psychological support as they consistently achieved higher rankings than many of the child’s human relationships, such as making one feel better about oneself, but not for practical problems children may have to face.

Furthermore qualitative study of early adolescents (10–14 years) found pet owners to have higher self-esteem than non-pet-owning peers amongst other pet-owning benefits such as friendship and stress reduction [14]. Importantly, a long term effect may be present; the self-concept of undergraduate students (14–49 years) was related to the age they were when they had their first pet [49]. Self-concept scores of undergraduate students were higher if participants were in early childhood (below 6 years old) (m = 349.42) or in adolescence (over 10 years old) (m = 361.81), than if they were in middle childhood (between 6 and 10 years old) (m = 342.14) when they owned their first pet.

The psycho-social wellbeing of youths due to goat ownership has been examined in Western Kenyan culture. A qualitative study using thematic analysis found that after orphaned 12–17-year-old children were given goats to care for, the development of pride, self-concept and self-worth was much improved due to goat ownership [50]. Owning goats, which are typically kept as property rather than pets, enabled children to create positive images of the self and of life, increased resilience and coping skills and increased social participation within the community. However, it must be recognised that goat ownership in this case may imply an increase in wealth therefore child welfare may not have been directly affected by interaction with the animals, but instead by an escape from poverty.

#### 3.1.4. Loneliness

Loneliness is likely a precursor for anxiety, depression and low self-esteem. There is some evidence that pet ownership may protect youths from loneliness and social isolation, and therefore may help to prevent depression. Pet-owning homeless youths aged 15–23 years reported fewer symptoms of both loneliness quantitatively (UCLA Loneliness Scale score of 1.8, compared to 2.3 among non-pet owners) [40] and qualitatively [44] than their non-pet owing peers in addition to reduced symptoms of depression. A large proportion of these youths had pet dogs (53%) and other companion animals, which they recognized as a coping strategy for loneliness due to their therapeutic nature and value [44].

The protective impact of pet ownership on loneliness has also been observed in less vulnerable populations. For example, high school students (13–19 years) who owned a pet reported significantly lower scores of loneliness (mean score of 33.7) than non-pet owners (39.5) using validated scales [45], regardless of ethnicity, gender, age, and family composition. In addition, loneliness scores were not affected by length of relationship with the pet or the number of pets owned. Companion animal attachment was positively related to the number of humans in the students’ social support network, suggesting that pet attachment may play an important role as a predictor. However, another study using validated measures of socio-emotional development of children aged 10–15 years found that pet owners were no more or less lonely than non-pet owners, although they did show a high degree of emotional closeness to their pets [42]. The impact of pet ownership on loneliness in younger children has not been investigated.

### 3.2. Behavioural Outcomes

There is mixed evidence on whether pet ownership affects behavioural outcomes in children or adolescents as shown in Figure 2. Amongst U.S. kindergarten children aged 5 years, perceived competence (cognitive competence, physical competence, peer acceptance and maternal acceptance) measured by parental report, was positively associated with pet attachment [19]. However, in the same study among older children (7 years and above), attachment to pets and perceived competence were generally unrelated. In a UK prospective follow up study, mixed equivocal findings were demonstrated in middle childhood (8–12 years). Findings suggest that behaviour improves when families first acquire a pet dog, but does not differ from non-dog-owning children longitudinally; dog owning children were reported to be less naughty, less argumentative, better behaved, and more co-operative by their mothers at the 1 month follow-up after acquiring a pet dog than non-dog owners, but there were no differences thereafter at the 6 and 12 months follow ups [48]. In addition, and perhaps surprisingly, caring behaviour was reported to decrease in dog-owning children in that study; however, it was not specified who, pets or humans, were the recipients of the caring behavior. Similarly, an American study of children in a paediatric primary care setting found no differences in the behaviour of dog owning children and non-dog owners aged 4–10 years measured by the Strengths and Difficulties Questionnaire [41]. In contrast, three other studies demonstrated how pet ownership increased behaviours of responsibility. Qualitative data from homeless youths suggests that dogs provide the opportunity to be responsible and care for another being, which in turn promoted healthier self-care choices and decision-making, for example, less alcohol consumption and improved financial choices [44]. Finally, a significant main effect was found (*p* = 0.006) for pet owners aged 8–13 years old showing greater autonomy (third grade m = 13.3, fourth grade m = 13.8, fifth grade m = 14.6, sixth grade m = 14.9) than non-pet-owning children (third grade m = 14.9, fourth grade m = 16.0, fifth grade m = 16.0, sixth grade m = 15.8) (lower mean indicative of greater autonomy). Explicitly, pet-owning individuals were more able to see their parents in roles other than the parental role and thus were deemed as more autonomous than non-pet owners [25]. The study suggested that pet ownership has the potential to foster the development of autonomous characteristics such as responsibility and self-reliance [25].

### 3.3. Cognitive Outcomes

Three studies have addressed the impact of pet ownership on child and/or adolescent cognitive development. A mixed methods thesis paper found that 10–14-year-old students with a stronger attachment to their pets had higher levels of validated social-cognitive development scores, for example in perspective-taking abilities, in comparison to students with a weak attachment to their pets (*p* < 0.001) [52]. However, no comparisons with non-pet owners were made. Pet care guidance also played a role; in the same study, students whose parents displayed more effective guidance of pet care showed stronger attachment with their pets (m = 28.19) than students who received less or no parental guidance on pet care at home (m = 14.28), and had more advanced skills of cognition and flexible problem-solving than students who received little or no guidance (*p* < 0.05) [52]. However, in a cognitive subscale of Attention (Pediatric Symptom Checklist 17) no differences were found when comparing dog-owning children to non-dog owners aged 4–10 years [41]. Lastly, research on companion animal bonding and young children’s social development found higher scores on parent reports of self-reliance and independent decision skills in strongly bonded pet-owning children compared to weak and moderately bonded pet-owning children, and non-pet-owning children (*p* < 0.05) [56].

### 3.4. Educational Outcomes

Four studies examined the impact of pets on educational outcomes. Pets may be useful in the engagement of both verbal and physical reciprocal behaviours. In a study investigating the effects of exposure to animals on children’s biological concepts, 2–6-year-old children with pets were more likely to attribute biological properties to animals than those without pets, and showed less anthropocentric patterns of extension of novel biological information, suggesting that having pets increases children’s knowledge of biology [53]. Thus, pet ownership could facilitate the development of a more sophisticated, human-inclusive representation of animals in children [53]. Similarly, 6–15-year-old children who owned two or more pets scored better on factual knowledge of animal anatomy than non-pet owners [54]. Furthermore, a Swedish study including qualitative interviews regarding the impact of pets on children’s development and desire to learn (“what can you learn from your pet?” and “What can your pet teach you?”) showed that owning dogs and cats may facilitate 4–5-year-old children’s learning and development process. Specifically, pet ownership aided the learning process in two sub-categories: 1. Developing empathy and emotions, and 2. Being good at school-related tasks [55]. Pets provided children with positive experiences and a sense of feeling good whilst increasing their knowledge of social behaviour. Exemplified sentiments expressed by many children in this study state “an animal listens only to you and gives you their full attention”. Such attention, in turn, may give children a sense of importance, satisfaction and a desire to learn more [55]. Finally, an early study of receptive vocabulary skills found bonding with a pet among 3–6-year-old children resulted in higher verbal intelligence scores in children moderately bonded to their pets (m = 124.20) in comparison to non-pet-owning children (m = 111.25) [56]. No research has been carried out to investigate the impact of pet ownership on later adolescent educational outcomes. 

### 3.5. Social Development Outcomes

The role of pet ownership and bonding with a pet among the social development of 3–6 year olds children has been evaluated by parental reports [56]. It was concluded that young children derive developmental benefits (social competence, empathy, and more positive attitudes toward pets) from their interaction with their companion animals. Bonding with pets appeared to be a stronger determinant of these associations than pet ownership. Taken together, children who bonded well with pets and children with better home environments had higher age-adjusted child development scores.

In contrast, one study showed that pet ownership might actually be detrimental to children’s social development, and may even reduce levels of social interaction in some children [48]. In a prospective study investigating the effects of obtaining new pet dogs, children’s attachment to pets at the 12 months follow up was associated with increases in the amount of time spent alone between baseline and 12 months (*p* < 0.05), and inversely associated to changes in children’s time spent with family (*p* < 0.05) and friends (*p* < 0.05), suggesting a that strong bond with a dog may result in less time spent with others. However, the study does not examine the quality of interactions; it cannot be assumed that quantity of time spent in social relationships with humans alone determines the quality of social interaction. A different study showed no evidence of an impact of dog ownership on social Externalizing outcomes (such as sharing and fighting behaviour, and understanding others feelings) in children aged 4–10 years [41]. Again, no effects of pet ownership on social measures were found in 13–18-year-old adolescents measured by the Pediatric Quality of Life Inventory which assesses social functioning and psychosocial health summary scores [43].

## 4. Discussion

The impact of pet ownership on child and adolescent development is a promising area of research but current evidence base does not permit firm conclusions. This paper provides a review of the evidence on the effects of pet ownership on emotional, behavioural, cognitive, educational and social development. Overall, the evidence suggests that pet ownership, and dog ownership in particular, may benefit these outcomes for children and adolescents. However, the evidence is mixed partly due to a broad range of different methodological approaches and varying quality of studies. In regards to the quality of the studies, the majority of the literature is categorised at low levels (levels 3 and 4) on the OCEBM criteria [39]. In addition, small samples sizes are common, and confounding factors have not always been accounted for. Therefore, the findings from which conclusions are drawn should be interpreted with caution.

**Figure 3:**
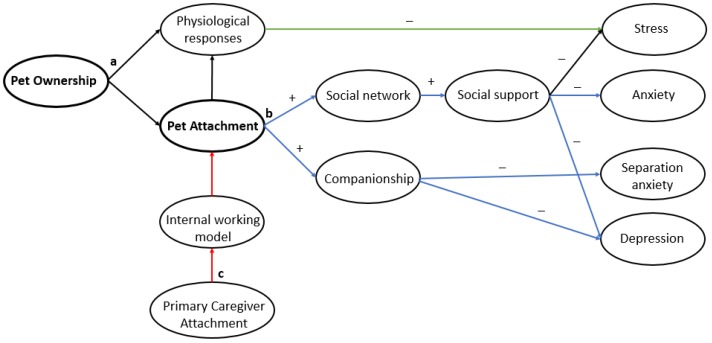
Hypothesized links for the impact of pet ownership and attachment on emotional health outcomes that postulates (**a**) physiological responses from pet interaction result in stress reduction (green pathway), and (**b**) anxiety, separation anxiety and depression are indirectly reduced by a wider social network and increased social support and companionship from pets (blue pathways) and (**c**) pet attachment may be indirectly affected by primary caregiver attachment (mother figure) through the internal working model (red pathway).

**Figure 4:**
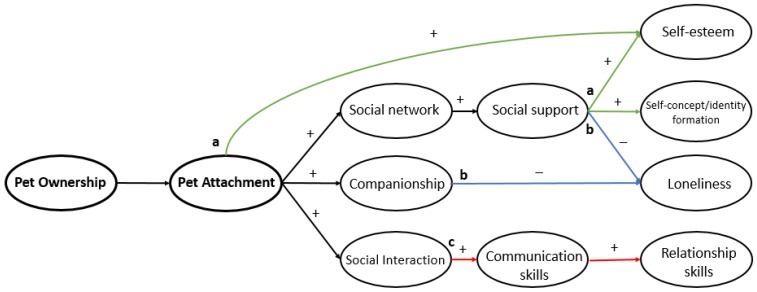
Hypothesized links for the impact of pet ownership and attachment on self-esteem, and loneliness that postulates (**a**) pet attachment directly increases self-esteem, and self-esteem and self-concept are increased indirectly through a wider social network resulting in increased social support (green) and (**b**) loneliness is reduced through a wider social network gained from having a pet, and increased social support and companionship from the pet (blue) and (**c**) relationship and communication skills are honed through increased social interaction (red).

**Figure 5:**
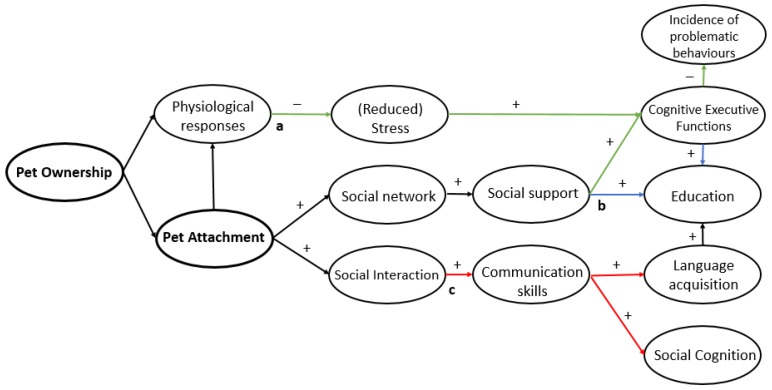
Hypothesized links for the impact of pet ownership and attachment on cognitive and educational outcomes, that postulates (**a**) Executive Functions are indirectly supported by stress reduction and increased social support, and therefore a reduced incidence of problematic behaviours follows (green) and (**b**) improved academic outcomes may result due to education being positively affected by improved executive functions and increased social support (blue) and (**c**) social cognition and language acquisition are enhanced by communication and social interaction with pets (red).

Diagrams have been conceptualized for the plausible relationships between pet ownership and children’s emotional, behavioural and cognitive outcomes (Figure 3, Figure 4 and Figure 5). These hypothesized diagrams focus strongly on the links found in the current literature within the field. We are well aware that the mechanisms behind these developmental processes are likely to be much more complex; they were simplified to focus on the plausible links found in this review, and for ease of interpretation. In addition, it is important to take into account the methodological issues, mixed results, and lack of replication of the literature used to postulate these hypothesized mechanisms. High quality research is needed to determine specific effects in pet type and child age, and to further explore if these links are truly causal. What follows is a brief summary of the results along with supporting research, followed by gaps in the literature and suggestions for further research directions. 

### 4.1. Emotional Outcomes

Overall, current evidence suggests that pet dogs may be beneficial in terms of preventing separation anxiety and social anxiety in both children and adolescents [41,57], however, this requires further investigation, as this finding is not consistent in older children and adolescents [42]. It is unknown whether pet dogs can reduce symptoms of anxiety in children. There is little evidence for any effects for other pet types. In regards to depression, there is a lack of research investigating the impact of pet ownership in youths, particularly in young children under 8 years old. Similar to anxiety, findings in depression seem to be varied. Findings may differ in younger age groups however, due to a typically higher level of interaction such as pet care and therefore stronger pet attachment [51]; the nature of the relationship with the animal may be important in conferring psychological benefits such as depression more likely than pet ownership. Overall it is suggested, but not conclusive, that vulnerable adolescents may benefit from pet ownership in terms of reduced depressive symptoms, and children who are attached to their dog during middle childhood may benefit in terms of resilience to depressive emotions in the long term. For young children, pet attachment seems to be a factor of importance for the prevention of depressive symptoms.

Within emotional health, the effect of pet ownership on child and adolescent self-esteem is currently the most studied outcome. Research generally demonstrated that children who grow up with companion animals showed higher levels of self-esteem and developed into more socially competent adults than children who do not grow up with companion animals [10]. Some studies found pet attachment to be a mediator of a relationship between self-esteem and pet ownership [47]; this is supported with longitudinal prospective research [48]. Therefore a relationship may exist between the level of attachment to one’s pet and self-esteem levels, similar to other components of psychological health. However, not all research is consistent with this suggestion; higher self-esteem and self-concept have been reported in pet owners irrespective of pet attachment [14,16,25] although causation cannot be implied here due to cross-sectional and qualitative study designs. Critical ages for the impact on pet ownership for self-esteem have been suggested [25]; pet ownership may have the greatest influence in children under 6 years old, and preadolescents and adolescents over 10 years old. Lastly, the majority of the evidence suggests that pets are useful in combating loneliness. Pet attachment was positively related to the number of humans in their social support network. This suggests pet attachment may again play an important role or, it could be that these people are better at forming attachments in general with humans and/or pets, but again due to study design, causation is not justified. The impact of pets on measures of loneliness in children under 10 years of age has not been investigated.

The significant findings in emotional health are consistent with research involving interaction with dogs as opposed to pet ownership, in 7–12-year-old children with insecure or disorganized attachment in stressful situations [58,59]. Dogs caused children’s cortisol levels to drop significantly faster and to lower levels after a stressor. It was concluded children with insecure and disorganized attachment may profit more in regulating their physiological stress levels from the interaction with a friendly dog than with a human or toy dog. The data suggest an important role of physical contact in the reduction of stress, although findings on the benefits of physical contact with companion animals are still generally unclear [60]. Further explanations behind why dog interaction and ownership may have such benefits for anxiety in youths center on the social catalyst effect [61], which states that pet dogs may stimulate conversation and alleviate social anxiety. Hormonal effects may also play a role; companionship and interaction with dogs can also lead to increased levels of oxytocin and reduced levels of cortisol, attenuating physiologic responses to stress and anxiety [17].

Importantly, child-dog interactions could prevent the evolution of emotional problems into full-fledged mental, emotional or behavioural disorders during adolescence or later life during adulthood [41], perhaps due to increased emotional support and resilience. This applies in particular to vulnerable (homeless) youths as companion animals provide emotional support in the form of loving relationships [40]. Furthermore pet therapy has the potential to reduce depressive symptoms and increase mood in children suffering from chronic physical illnesses such as haematological and oncological disorders, cystic fibrosis, diabetes, transplants, and other medical disorders [62]. Further research is needed as to whether childhood pet ownership may have similar effects.

Both quantitative and qualitative research find self-importance to be a common theme; pets act as a form of psychological support by making youths feel good about themselves and are enabled to create positive images of the self [15,16]; this also applies to non-western cultures [50]. These findings are promising and suggest that pet ownership should be investigated as a strategy to increase self-esteem in developing youths. Findings that support this include research carried out using a horse therapy program; although no intervention effect was found on self-esteem, an increase was found in perceived social support in comparison with the control group [63]. Pets such as horses and dogs are most likely to increase social circles and the number of human contacts, and if so, could increase emotional health outcomes such as self-worth and self-esteem. Overall the current research generally displays potential for pets to increase children and adolescents’ resilience and self-worth. In particular, adolescent loneliness and isolation is an important issue, and if untended can manifest as a host of various physical and emotional problems, including anxiety, depression and low self-esteem [64] and poor academic achievement [65]. Companion animals are used as a coping strategy for loneliness in youths due to their therapeutic nature [44]. It is possible that companion animals offer a reciprocal affectionate and non-judgemental relationship, which has obvious benefits for child and adolescent development. Notably, it is difficult to unravel other variables that may explain why pet owning youths seem to appear less lonely. The importance of parenting styles has previously been suggested [14], which may differ in pet owning families, and is likely to increase responsibility, autonomy, empathy and socialization in comparison to non-pet owning households. However, pet ownership may independently impact on the development of empathy and socialization without the influence of parenting style; it is plausible that parents who keep household pets are actually fostering these qualities by proxy [45], therefore lessening childhood loneliness. Further well-designed studies are recommended for additional clarity, to infer causality, and to conclude whether there is a link between companion animals and child and adolescent loneliness. 

### 4.2. Behavioural Outcomes

The evidence is mixed for the impact of pet ownership on child and adolescent behavioural outcomes. Results of different research studies are not consistent on whether perceived competence in children is positively and significantly associated with pet ownership and/or attachment, dependent on age [19]. There appears to be no long-term behavioural benefit from acquiring a pet dog, as child behaviour only improves when families first acquire the dog [41,48]. Nevertheless, there is literature to suggest that pet ownership and pet care in particular is associated with increases in positive behaviours such as responsibility [10,25,44,45,66]. Therefore pet ownership and pet care responsibilities may encourage positive behavioural development in terms of independence, and other autonomous characteristics such as self-reliance [25]. Further well designed research is needed using objective measures of behaviour, such as school reports. In addition, as child and adolescent behaviour can predict future educational attainment [67], it would be interesting to explore the potential links between pet ownership, behavioural outcomes and other indirect developmental relationships. Other non-experimental mechanism-based reasoning reports suggest that pet owning children are likely to show decreased violence and antisocial behaviours, as pet ownership has positive effects of a wide range of developmental outcomes including social and moral development [68]. However, no evidence of this was found in studies reviewed here. The idea that childhood and adolescent behaviour may predict future antisocial activity is not new. Childhood disruptive behaviour has powerful long-term effects on adult antisocial outcomes, which continue into middle adulthood [69]. If pets can promote such positive behaviour, they may be involved in early interventions. However, there is very little research in the area, and there are findings to argue against this claim; among youth offenders childhood bonding with a pet was not related to antisocial personality traits [70].

### 4.3. Cognitive Outcomes

Pet ownership, attachment and parental pet care guidance were associated with higher levels of some areas of social cognitive development for example perspective taking abilities, and cognitive flexible problem solving skills [52]. Furthermore, self-reliance and independent decision skills were higher in pet-owning children compared to children who do not have pets [56]. However, other areas of cognition were not affected in a similar manner; no differences in attention were found in dog owning children compared to non-dog owners [41]. Caution must be taken when interpreting findings. In addition to their inability to establish causality, most studies inadequately controlled for potential confounding factors. It cannot be concluded pet care guidance increases cognitive function with respect to higher level thinking and flexible problem solving. These higher cognitive skills may instead be due to good parental guidance in general rather than pet care guidance. Other important confounding factors also need to be ruled out such as the quality of children’s home environments, beyond the presence of animals, which has been linked with both the concurrent and longitudinal cognitive development of preschool children [28,56,71].

Current research advocates pet ownership and animal interaction as a catalyst for learning and progressing in both cognitive and psychosocial domains [29,30,31,32,33]. The mechanisms behind the influence of pet interaction on cognitive development are not fully understood. Speculations include improved cognitive Executive Functions (EFs) through stress reduction and social support which in turn can positively affect behaviour and academic outcomes [38] however, this remains to be tested. Research has suggested that pets may aid a quicker progression of the four major periods of cognitive development [72] (sensorimotor stage, preoperational stage, stage of concrete operations, and the formal operation stage [73]) however, further study is warranted. As animals are “predictably unpredictable” [9], pet behaviour to the observing child represents what cognitive development theory [73] argues is the route of all learning, namely, cognitive incongruity, moderate discrepancy from established schema, and novel information [10] however, this statement does not take into account that pet behaviour varies greatly and remains to be tested empirically. Younger children (i.e., children in the preoperational stage) may be beginning to learn and develop their concept of social relationships, and interacting with pets may promote young children’s cognitive development; existing research appears to support this idea [52,72]. Introducing children to animals during such a sensitive period may produce optimal results in terms of promoting their abilities to enhance social cognitive development [52], in particular perspective taking abilities, although more empirical research is needed to infer this. Possible mechanisms may include pet ownership enhancing the progression of the child’s internal thinking (i.e., reorganization and advancement) which shapes their schema and may enhance overall cognitive development. In addition, as children include their pets in physical, imaginative, and free play [72], social and cognitive functioning may be enhanced due to practicing problem solving abilities and creativity [74]. Other than social-cognition, further well-designed research is required on pet ownership that examines mainstream cognitive outcomes such as executive function, memory and IQ.

### 4.4. Educational Outcomes

Pets have the potential to improve educational outcomes. For many children, companion animals are likely powerful motivators for learning [9] and development [9,55,75]. Pets have also been found to enhance performance in school-related tasks [55] and enrich children’s vocabulary [56]. Although mechanisms are not clear, this is possibly due to children learning and retaining more about subjects they are emotionally invested in, and furthermore learning is optimized when it occurs within meaningful relationships. Pets also engage children in both verbal and physical reciprocal behaviours [53]. Interestingly, research has demonstrated that pet owners benefit from more advanced biological knowledge than non-pet owning children suggesting that pets facilitate the development of a more sophisticated, human-inclusive representation of animals, knowledge about the internal structure of animals and factual anatomy [53,54]. So far, no research has investigated the impact of pets on later adolescent educational outcomes. The support of pets in children’s learning process is also demonstrated in research involving classroom animals with respect to reading skills [35,36], social functioning and academic competence [37], emotional stability within school and attitudes towards school [76]. The evidence base is strongest for dogs; the presence of a dog in the classroom has been shown to help children exercise better cognitive executive functions and perform better academically [77]. Further research is required to investigate whether pet ownership is associated with academic attainment.

### 4.5. Social Development Outcomes

Findings are mixed in terms of the impact of pet ownership on children’s social and socio-emotional development. Childhood pet ownership encourages healthy social development in terms of social competence, social networks, social interaction, social communication, empathy and social play behaviour, leading to higher age-adjusted developmental scores [10,56,68]. However, it must be noted that pet bonding and, therefore, pet attachment appeared to be a stronger determinant of these benefits than pet ownership [56]. The finding that pets increase social networks is encouraging; how a child develops is strongly influenced by the child’s social network, for example the support provided by social networks can enhance self-esteem and contribute to mental health, by providing a buffering, protective function against psychosocial stress [78]. In addition, the finding that pets increase social play behaviour and communication is important, and strongly suggests that pets have the potential to encourage the development of effective socially interactive relationships with others. Alternatively, pets might actually be detrimental to social development and may even reduce levels of social interaction with family and friends in some children [48] which is likely due to the child substituting human contact for interaction with their pet. However, the reduced quantity of social interaction does not mean the quality of these human relationships will suffer. In addition, no significant effects were found on the impact of childhood dog ownership on social externalizing outcomes (such as sharing, fighting and understanding others’ feelings) [41], nor social functioning in adolescents [43]. Other research finds social provisions in children are enhanced by classroom pets with children displaying more prosocial behaviours with peers [37]. Further high-quality research is needed to infer causality. In addition the majority of the research has been conducted when interactions on social media were not yet very common. Children’s experience of “expanded” social networks is very different now than it was a couple of years or decades ago. As more and more children experience friendships (and abuse) online and on social media, the effects of pets on the feelings of social isolation in this context would be particularly cogent.

### 4.6. Risks/Costs to Children and Adolescents Associated with Pet Ownership

Along with the benefits of the ownership of companion animals, which may include improved child behaviour and development, certain negative consequences have been noted. These include zoonotic infections [79], allergy and asthma [80], bites and other injuries [11] and the psychological and emotional costs due to pet bereavement [81]. Young children are at a greater risk of zoonotic infection; this is a particular concern for immunocompromised children (reviewed in [82]). In addition, children are at a greater risk of animal bites from a household pet (e.g., about 72%–80% of children are bitten by a familiar dog [83,84,85]). Children under 5 years of age are significantly more likely than older children to provoke animals before being bitten and are most at risk of serious injury [83,84,86,87]. 

### 4.7. Methodological Limitations

The review reveals mixed evidence and conflicting results. In studies investigating pet ownership on human health and development such inconsistent findings are not infrequent due to use of a wide diversity of designs, small effect sizes and small and homogeneous self-selected samples [88,89]. In addition the research findings within the field are often limited by lack of replication [90]. 

This review highlights a number of particular methodological limitations that require addressing in future studies. If these concerns are addressed, then the research quality in the field will be significantly improved. Firstly, there is inconsistency in how studies classify non pet owners. The studies reported here did not appear go into any detail regarding comparators; for example youths with recently deceased pets are likely to be regarded as non-pet-owners. Papers commonly specify non pet owners as “non-dog” and “non-cat” owners, however, this frequently fails to account for potential effects of other companion animals on the outcomes of interest. Pet owners are often treated as one homogenous population without consideration of differences between them or of differences in species owned, their attitudes to pet ownership and pet attachment, both of which are likely to impact potential benefits from their interaction with their pets. Secondly, in some studies, the reliance of subjective self-reported data in place of objective validated outcomes is problematic, due to an increased probability of false negative and false positive reporting. 

Thirdly, the majority of studies to date have been cross-sectional, which means that the direction of the association between pet ownership and different aspects of child development cannot be determined. For example, children deemed by their parents as more responsible may be viewed as more ready to take on the role of pet owners, and therefore, more likely to get a pet than children who are viewed as less responsible or mature. This reverse causality could still result in a positive association between pet ownership and responsible behavior, but in this case, responsible behavior would cause pet ownership and not the other way around. Due to the nature of the independent variable (owning a pet or not), research in this field cannot be truly experimental, and therefore prospective studies are needed to determine the temporal direction between pet ownership and the outcomes [10,25].

Fourth, longitudinal and prospective studies in pet ownership and child development are needed to determine the long-term consequences for children of establishing relationships with pets and other animals. A lack of longitudinal and epidemiological data in this area hampers the development of appropriate and effective interventions [89].

Fifth, research into the effects of animals on human health and development have also been historically weak in terms of statistical power and the ability to appropriately control for confounding variables [90]. Pet ownership has been associated with numerous socio-demographic factors [6,7,91,92,93]; the majority of studies in this review have failed to take into account some of these factors. Conflicting findings may be due, at least in part, to the inadequate control of variables identified as potential confounders. Furthermore, a child’s interaction with pets is mediated by interactions with adults, siblings, and peers. Therefore, a life-course approach is needed to specify mediational models and pathways to later developmental, and to understand the different forms of social and emotional support pets may provide, as well as how this support is contextualized within adult, peer and pet relationships over time [66,89]. For example, a pet may positively influence emotional and mental health of both children and adults within a family unit. Because of the reciprocal nature of all relationships, children who show more positive behavior due to bonding with their pet, may elicit more positive responses from their parents, thus contributing to an overall positive family functioning. In turn, parents, who benefit from lower levels of anxiety or depressive symptoms from owning the same pet, may interact more positively with their children.

Another important limitation for the majority of studies included in the review is that it is not possible to know whether families with children having no or minimal challenges with emotional health or general developmental difficulties are more or less likely to live with companion animals, compared with families with children having challenges.

Last, it is possible that the published literature on the impact of pets on children’s health is biased by selective publication of positive results. For example, studies demonstrating a significant effect of pet ownership may be more likely to be published and cited by others than studies with negative findings. The lack of negative/null findings illustrated in Figure 2 suggests a high likelihood of this “file drawer effect,” which may skew the available scientific literature on human-animal relationships [90].

## 5. Conclusions

In summary, current evidence suggests that overall, pet ownership may be beneficial to child and adolescent emotional, cognitive, behavioural, educational and social development. Although the majority of studies performed to date had methodological weaknesses, the pattern of findings among sub-populations and age groups suggests that companion animals have the potential to promote and contribute to healthy child and adolescent development. However, there is a scarcity of research to elucidate the mechanisms through which pet ownership promotes child development. This is required to identify the processes that underlie the observed relationship between pet ownership, pet attachment and child development. Future research should examine the potential effects of different pet types. Although the majority of research has taken into account the types of pets children owned, dogs appear to be the most researched and beneficial, perhaps due to a higher level of interaction and reciprocation in comparison to other pets. There is little understanding so far of potentially differential effects of different types of pets on specific psychological, behavioural, and social problems [94]. Further research is required to investigate the mechanisms through which pet ownership promotes child and adolescent development. Future studies must better account for confounding variables, and preferably use longitudinal and as strictly controlled designs as possible in order to infer causality.

## Figures and Tables

**Table 1:** Oxford Centre for evidence-based medicine 2011 levels of evidence.

Level of Evidence	Description
Level I	Systematic review of Randomized Controlled Trials
Level II	Randomized Trials
Level III	Non-randomized controlled cohort/follow-up studies
Level IV	Case-series, case-control studies
Level V	Expert opinion/Mechanism-based reasoning

Level I = highest evidence (lowest potential for bias); Level V = lowest evidence (greatest potential for bias).

**Table 2:** Evidence for the impact pets have on child and adolescent development.

Reference No.	Topic	First Author (Year)	OCEBM Level (2011)	Type of Animal	Sample Size	Participant Age	Participant Gender	Study Type/Design	Confounding Considered?	Outcome
[40]	Emotional health (depression)	Rhoades (2015)	IV	Dog (53%), cat (22%), hamster, rat, chinchilla, fish, iguana	332	13 years	91 female 234 male	Cross-sectional survey Control group used.	Yes	Pet owning homeless youths reported fewer symptoms of depression and loneliness than their non-pet owning peers.
[41]	Emotional health/behavioural/social/ cognitive development	Gadomski (2015)	IV	Dog	643	4–10 years	289 female 354 male	Cross-sectional survey Control group used	Yes	Having a pet dog in the home was associated with a decreased probability of childhood anxiety in some components (panic, social and separation anxiety) of the SCARED-5 (Screen for Child Anxiety Related Emotional Disorders). However, no difference was found between dog owning and non-dog owning children in their histories of mental health problems. Nor were there significant effects of pet ownership in childhood social, emotional, and behavioural development.
[42]	Emotional health (loneliness, attachment, social anxiety)	Vidovic (1999)	IV	Dog (26.2%) Cat (9.2%) Other (19.0%)	826	10–15 years	425 female 401 male	Cross- sectional, correlational design Control group used	No	Children who scored higher than average on the attachment to pets scale showed significantly higher scores on empathy and prosocial orientation scales. Pet owners, regardless of age, were not significantly lonelier than non-owners, nor were they socially more anxious.
[43]	Emotional health	Mathers (2010)	III	Dog, Cat, Horse or Pony and Other	928	13–19 years	460 female 466 male	Cross-sectional data from longitudinal school-based population study	Yes	Neither owning a pet nor time spent caring for/playing with a pet appeared to be related to better adolescent emotional health, social development or well-being. Neither did they contribute to negative outcomes. These findings may not apply to other (younger) age groups with a typically higher level of interaction with their pets.
[44]	Emotional health (loneliness)	Rew (2000)	IV	All	32 10	16–23 years 15–23 years	14 female 18 male 3 female 6 male 1 “both”	Qualitative focus groups Qualitative interviews	No	Dogs or animal companions are used as a coping strategy for loneliness. Vulnerable adolescents who are homeless often recognize the therapeutic value of pets.
[45]	Emotional health (loneliness, social support)	Black (2012)	IV	Dogs (67%), Cats (18%), Horses (5%) Rodents and Reptiles (10%)	293	13–19 years	158 female 135 male	Cross-sectional survey Control group used	No	High school student pet owners reported less loneliness than non-pet owners. Companion animal attachment was positively related to the numbers in the social support network.
[46]	Emotional health (self-esteem)	Arambasic (1999)	IV	Dog, cat and other (birds, fish, rodents and turtles)	612	11–15 years	311 female 301 male	Cross-sectional survey Control group used	Yes	Pet ownership had no significant impact on the self-esteem of war-traumatized children. Self-esteem of pet owners did not differ from self-esteem of non-pet owners, and the type of pet owned also had no effect on self-esteem.
[25]	Emotional health (self-esteem, self-concept)	Van Houtte (1995)	IV	All	130	8–13 years	59 female 71 male	Cross-sectional survey Control group used	Yes	Higher self-esteem was reported in pet owners than in non-pet owners, as was a higher autonomy, and self-concept. Attachment to animals was not found to be higher in the pet-owning group and greater attachment to animals was not found to be related to higher scores on the dependent measures.
[16]	Emotional health (self-esteem)	Bryant (1990)	IV	All	213	8–13 years	Not reported	Qualitative interviews Principal component factor analysis	No	Children felt their companion animals benefited them in 4 factors: (1) mutuality (reciprocity in the caring and loving between pet and child); (2) enduring affection (even if the child misbehaves the pet will still love him or her); (3) self-enhancing affection (the child–pet relationship is perceived by children as one that makes them feel good about themselves and imparts a sense of importance) and (4) exclusivity of the child–pet relationship
[47]	Emotional health (self-esteem)	Triebenbacher (1998)	IV	All	436	9–18 years	204 female 232 male	Cross-sectional survey Control group used	No	No direct relationship between levels of self-esteem and pet ownership in school children. An indirect relationship was found between pet ownership and self-esteem mediated by attachment to companion animals. As with other components of psychological health, there may be a relationship between levels of attachment to one’s pet and self-esteem benefits accrued.
[15]	Emotional health (self-esteem/social support)	McNicholas (2001)	IV	All	22	7–8 years	9 female 13 male	Qualitative interviews	No	Pets were often ranked higher than certain kinds of human relationship, and featured prominently as providers of comfort, esteem support and confidantes for a secret. Dogs and cats offer special relationships for provision of psychological forms of support but not for the more practical problems a child might have to deal with. The fact that cats and dogs frequently ranked higher than many human relationships suggests the value that children place on their pets and the functions they serve.
[48]	Emotional health (confidence, tearfulness, self-esteem)	Paul (1996)	III	Dog	56	8–12 years	27 female 29 male	Prospective questionnaire survey Control group used	Yes	Higher levels of attachment to the dog were positively associated with changes in confidence by the 6 month follow-up, and negatively associated with changes in tearfulness or weepiness by the 12 months follow-up. The positive association between dog attachment and subject children’s confidence (at the 6 months follow-up) and its negative association with tearfulness (at the 12 months follow-up) were more consistent with the findings of previous studies which suggest that pet keeping can be associated with higher levels of self-esteem in some children
[14]	Emotional health (self-esteem/stress)	Covert (1985)	IV	All	285	10–14 years	Not reported	Qualitative Interview Mixed methods	No	Early adolescent animal owners had higher self-esteem than non-animal owners. Adolescents felt they gained responsibility (rabbit/hamster), and friendship/love/fun (dog, horse and fish/bird) from pet ownership. Early adolescents used pets for stress reduction.
[49]	Emotional health (self-concept)	Poresky (1988)	IV	All	188	Undergraduate students 14–49 years	99 female 89 male	Cross-sectional survey	No	Self-concepts of undergraduates were related to the age when they had their first pet. Total Positive Self-Concept scores were higher if participants were under 6 years or over 10 years old than if they were between 6 and 10 years old when they had their 1st pet. Similar results were found for the social subscales.
[50]	Emotional health (self-concept and psychosocial development)	Winsor (2011)	IV	Goat	15	12–17 years	7 female 8 male	Qualitative interviews	No	Goat ownership enabled children to create positive images of self and life—deriving emotional benefits. Goat ownership provides orphaned and vulnerable children with opportunities for positive social participation and community engagement that can facilitate children’s resilience and wellbeing.
[51]	Emotional health (psychosocial development)	Davis (1987)	IV	Dog	22	10–12 years	13 female 9 male	Cross-sectional survey	No	Reasons for acquiring a dog centred on the companionship and emotional dimensions of pet ownership. It appears that the preadolescent does not actually assume a large proportion of daily, routine pet care responsibility, instead they acquire a pet dog for companionship and emotional dimensions of pet ownership.
[52]	Cognitive development	Maruyama (2011)	IV	All	65	10–14 years	43 female 22 male	Mixed methods Cross-sectional survey Qualitative interviews	No	Students who showed stronger attachment with their pets had higher levels of social cognitive development than students who showed weaker attachment with their pets. Students whose parents show more effective guidance on pet care have more advanced skills of thinking and solving problems in flexible manner than students who do not receive any or less guidance on pet care at home.
[53]	Educational (biological knowledge/psychological reasoning)	Geerdts (2015)	IV	Dog and Cat	24 96	2–6 years	15 female 9 male	Observations, cross-sectional survey and experimental tasks	No	Both 3 and 5-year-olds with pets were more likely to attribute biological properties to animals than those without pets. Both older and younger children with pets showed less anthropocentric patterns of extension of novel biological information. The results suggest that having pets may facilitate the development of a more sophisticated, human-inclusive representation of animals.
[54]	Educational (biological knowledge)	Prokop (2008)	IV	All	1541	6–15 years	753 female 788 male	Experimental task	Yes	Experiences with rearing pets significantly contributed to children’s knowledge about animal’s internal organs. Children who reported keeping 2 or more animals acquired better scores than children keeping only 1 or no animals.
[55]	Educational/ Emotional health	Svensson (2014)	IV	Dog and Cat	24	4–5 years	12 female 12 male	Qualitative interviews	No	The pet supports the child in the learning and development process by (l) Developing empathy and emotions; (2) Being good at school-related tasks. Pets provide children with positive experiences and a sense of feeling good.
[56]	Social development/educational/ cognitive development	Poresky (1989)	IV	All	88	3–6 years	Not reported	Cross-sectional survey /interview	Yes	Developmental benefits were primarily in the children’s social domain including social competence, empathy, and pet attitudes. “Pet bonding“ appeared to be a stronger determinant of the pet associated benefits than “pet ownership“. Children with companion animals and a better home environment showed higher age-adjusted child development scores. Intellectual development benefits were also associated with the strength of the bond between the child and his/her pet. Self- reliance and independent decision skills were higher in the children who have pets.
[19]	Socio-emotional/ behavioural development	Melson (1991)	IV	All	120	5, 7, 10 years	Not reported	Cross- sectional survey/ individual interview	No	Among kindergarten children, perceived competence was positively and significantly associated with diverse dimensions of attachment to the pet. This was not found in older children. Pet attachment was higher for older children and those whose mothers were employed.
